# A model of perceptual discrimination under sequential sensory evidence

**DOI:** 10.1186/1471-2202-14-S1-P102

**Published:** 2013-07-08

**Authors:** Etienne Hugues, Carlos Stein Naves de Brito, Wulfram Gerstner, Ranulfo Romo, Gustavo Deco

**Affiliations:** 1 Center of Brain and Cognition, Department of Information and Communication Technologies, Universitat Pompeu Fabra, Barcelona, 08018, Spain; 2 School of Computer and Communication Sciences and Brain Mind Institute, School of Life Sciences, Ecole Polytechnique Fédérale de Lausanne, Lausanne, 1015, Switzerland; 3 Instituto de Fisiología Celular-Neurociencias, Universidad Nacional Autónoma de México, México, 04510, Mexico; 4 Institució Catalana de Recerca i Estudis Avançats, Universitat Pompeu Fabra, Barcelona, 08018, Spain

## 

Perceptual discrimination may be interpreted as a decision between alternatives based on available sensory evidence. In many experiments, the different alternatives are encoded by quite distinct neuronal groups. In this case, proposed neural models consider that the decision results from the competition between decision-specific neuronal groups, each of these integrating distinct sensory evidence [1]. Alternatively, evidence may be presented in a sequential manner, and the different stimuli may be encoded by the same neuronal group, as exemplified by experiments where monkeys are engaged in a vibrotactile discrimination task [2]. To achieve discrimination in this case, the nervous system needs to keep a trace of the previously presented stimuli. How the correct discrimination can be learned and implemented is poorly understood.

To address these questions, we use a modeling approach. We concentrate on the particular case of the vibrotactile discrimination task on which experimental insight has been accumulated over the last years [2]. The partial differential (PD) neurons in monkey area VPC, encoding both sequentially presented vibrotactile stimuli (with frequencies f_1 _and f_2_) by keeping the memory of the first one during a delay period, have been reproduced in a spiking neuron network model with short-term facilitating synapses [3]. We want to explore how these PD neurons may be used to discriminate between both stimuli configurations: f_1 _> f_2 _or f_1 _< f_2_. Based on the experimental evidence, we model a heterogeneous PD neuronal population, encoding both frequencies in multiple ways. Downstream to the first network, we add a competition-based decision making spiking neuron network [1]. To obtain the desired neural dynamics of the coupled two-networks model, we choose the model parameters using a simplified mean-field description of the model. To make the best possible decisions, the strengths of the synapses projecting from the PD neurons to the decision neurons must be learned. Based on reinforcement learning theory, we use a learning rule which maximizes reward. It depends on a reward prediction error, evaluated using the reward history. We instantiate this rule using a reward based spike-timing-dependent plasticity [4]. Learning takes place after the second stimulus presentation. We find that the task can be efficiently learned for any number of PD neuron types, even when their stimulus encoding function is nonlinear and noisy.

In conclusion, the present biologically plausible two-networks model is able to solve a perceptual discrimination task under sequential sensory evidence.
